# Erythrocyte-binding assays reveal higher binding of *Plasmodium knowlesi* Duffy binding protein to human Fy^a+/b+^ erythrocytes than to Fy^a+/b-^ erythrocytes

**DOI:** 10.1186/s13071-018-3118-8

**Published:** 2018-09-26

**Authors:** Mun Yik Fong, Fei Wen Cheong, Yee Ling Lau

**Affiliations:** Department of Parasitology, Faculty of Medicine, University of Malaya, Kuala Lumpur, Malaysia

**Keywords:** *Plasmodium knowlesi*, Duffy binding protein, Duffy antigen, Erythrocyte-binding assay

## Abstract

**Background:**

The merozoite of the zoonotic *Plasmodium knowlesi* invades human erythrocytes *via* the binding of its Duffy binding protein (PkDBPαII) to the Duffy antigen on the eythrocytes. The Duffy antigen has two immunologically distinct forms, Fy^a^ and Fy^b^. In this study, the erythrocyte-binding assay was used to quantitatively determine and compare the binding level of PkDBPαII to Fy^a+/b+^ and Fy^a+/b-^ human erythrocytes.

**Results:**

In the erythrocyte-binding assay, binding level was determined by scoring the number of rosettes that were formed by erythrocytes surrounding transfected mammalian COS-7 cells which expressed PkDBPαII. The assay result revealed a significant difference in the binding level. The number of rosettes scored for Fy^a+/b+^ was 1.64-fold higher than that of Fy^a+/b-^ (155.50 ± 34.32 and 94.75 ± 23.16 rosettes, respectively; *t*_(6)_ = -2.935, *P* = 0.026).

**Conclusions:**

The erythrocyte-binding assay provided a simple approach to quantitatively determine the binding level of PkDBPαII to the erythrocyte Duffy antigen. Using this assay, PkDBPαII was found to display higher binding to Fy^a+/b+^ erythrocytes than to Fy^a+/b-^ erythrocytes.

## Background

Almost 15 years ago, *Plasmodium knowlesi*, a malaria parasite of long-tailed and pig-tailed macaques in Southeast Asia, was reported to cause a high number of human infections in Sarawak on Borneo Island [1]. Since this landmark report, human knowlesi malaria has been encountered in other parts of Borneo Island, Peninsular Malaysia, all countries in Southeast Asia, the Andaman and Nicobar Islands of India, and southwest China [2, 3]. In addition, knowlesi malaria has been reported in ecotourism travellers from the USA, Europe, the Far East and Australasia, who visited the forests of Southeast Asia [4]. Hitherto, more than 4000 cases of human knowlesi malaria have been reported in Malaysia and *P. knowlesi* has overtaken *Plasmodium vivax* (a human malaria parasite) as the main cause of malaria in the country.

Invasion of the malaria parasite merozoite into its host erythrocyte is a multi-step process. It starts with the attachment of the merozoite to the erythrocyte surface, followed by apical reorientation of the merozoite, formation of a tight-junction between the cellular membrane of the merozoite and erythrocyte, and entry of the merozoite into the erythrocyte cytosol to form a parasitophorous vacuole, within which the parasite grows and replicates [5]. The tight-junction formation is a key step of the invasion, and it involves interaction between the merozoite’s binding protein and its corresponding receptor on the surface of the erythrocyte. In the case of *P. knowlesi*, the interaction occurs between its Duffy binding protein (PkDBP) and the erythrocyte’s Duffy antigen receptor for chemokines (DARC) [6]. PkDBP is a large protein and can be divided into seven regions (I-VII). The amino acid motifs for binding to the erythrocyte DARC reside in region II. PkDBP is encoded by an α-gene and therefore region II is known as PkDBPαII. The human erythrocyte Duffy (Fy) antigen has two immunologically distinct forms. These forms, known as Fy^a^ and Fy^b^, are the result of a single amino acid substitution [7] in the domain that binds with PkDBPαII.

Similar to *P. knowlesi*, *P. vivax* also uses the Duffy antigen receptor to invade erythrocytes. Recent studies have shown that Fy^a^, compared with Fy^b^, significantly reduced the binding of *P. vivax* Duffy binding protein (PvDBP) at the erythrocyte surface. Interestingly, this is associated with a reduced risk of vivax malaria in humans [8]. A limited number of studies have been carried out to measure and compare the binding level of PkDBP to the Fy antigen. A study conducted in the late 1980s indicated greater binding of a 135 kD protein, presumably PkDBP, to Fy^b^ than to Fy^a^ [9]. However, the experiments in the study used metabolically labeled proteins in the supernatant of *in vitro* cultured *P. knowlesi*, and the binding activity was qualitatively determined by probing the 135 kD protein with *in situ* erythrocytes and electrophorectically-separated erythrocyte proteins on blots.

The *in vitro* erythrocyte-binding assay is widely used in studies on binding activity between malaria parasites’ erythrocyte binding proteins and their corresponding receptors on the host erythrocyte. In this assay, the protein of interest is heterologously expressed on the surface of mammalian cells. Erythrocytes are added to the cells and positive binding is shown by the formation of rosettes, which are aggregates of erythrocytes surrounding cells expressing the protein. The advantage of this assay is that binding level can be quantitatively determined by counting the number of rosettes [10–12]. Hence in the present study, this assay was used to compare the binding level of PkDBPαII to Fy^a+/b-^ and Fy^a+/b+^ erythrocytes, the two common Duffy phenotypes in the Malaysian population.

## Methods

### Construction of recombinant plasmid PkDBPαII for a eukaryotic expression system

Construction of recombinant plasmid PkDBPαII for a eukaryotic expression system was performed as previously described [12]. Briefly, the *PkDBPαII* gene was amplified from the *P. knowlesi* genomic DNA extracted from patient blood by PCR, and then cloned into PCR vector pGEMT® (Promega Corp, Wisconsin, USA). Recombinant PkDBPαII-pGEMT® plasmid was digested using restriction enzyme *Bgl*II and cloned into the eukaryotic expression vector pDisplay™ (with fluorescent reporter gene *GFP* at the C-terminus) *via* multiple cloning steps. The recombinant PkDBPαII-pDisplay™ plasmid was extracted and the concentration of the plasmid was determined prior to transfection into COS-7 mammalian cells.

### Expression of PkDBPαII on COS-7 mammalian cells

COS-7 (ATCC® CRL-1651™) cells were grown in a 5% CO_2_ incubator at 37 °C using DMEM (Thermo Fisher Scientific, Waltham, Massachusetts, USA) complete medium supplemented with 1% penicillin-streptomycin and 10% fetal bovine serum (FBS). Prior to transfection, the COS-7 cells were plated into 6-well culture plates. When the cells reached > 90% confluency, transfection was performed with 2.5 μg of PkDBPαII-pDisplay™ plasmid DNA using Lipofectamine 3000 reagent (Invitrogen, Carlsbad, CA, USA). Serum-free DMEM incomplete medium was used during transfection and the cells were grown in a 5% CO_2_ incubator at 37 °C. At 24 h post-transfection, the culture medium was replaced with DMEM complete medium containing 10% FBS, and the cells were further incubated for another 24 h. These transfected COS-7 cells were then used in the erythrocyte-binding assay.

### Detection of rosette formation in the erythrocyte-binding assay

Blood donors were selected and the Duffy genotype of their erythrocytes was confirmed using allelic specific PCR (ASP-PCR) as previously described [13]. One day before the assay, erythrocytes were collected from the blood donors. The erythrocytes were washed 3–4 times with incomplete DMEM and the washed erythrocytes were stored at 4 °C prior to use. At 48 h post-transfection, PkDBPαII protein was expected to be expressed on the surface of the COS-7 cells. Washed erythrocytes were diluted to 1% haematocrit using incomplete DMEM and added into wells with transfected COS-7 cells, followed by 2 h incubation at 37 °C. Erythrocytes were discarded and the wells were washed 3 times with 1× PBS to remove non-binding erythrocytes. The wells were incubated with 1 μg/ml Hoechst 33342 dye (Invitrogen) for 1 min in darkness to stain the nuclei of COS-7 cells. After three washes with 1× PBS, 1% paraformaldehyde was added and incubated at room temperature for 10 min to stabilize the rosettes. All washes were carried out in a gentle manner to prevent formed rosettes from being dislodged. Nikon Eclipse TE300 inverted fluorescence microscope was used to observe the successfully transfected COS-7 cells under a FITC-filter (488 nm excitation wavelength) and to determine the number of rosettes. Rosettes were counted as positive when adherent erythrocytes covered more than 50% of the COS-7 cell surface [10, 11], whereas binding was scored as negative when no rosette was observed in the entire well. The number of rosettes in 30 fields at a magnification of 200× was counted to score the binding. Four Fy^a+/b-^ and four Fy^a+/b+^ blood samples were used in the assay. Triplicates were performed for each blood sample. COS-7 cells transfected with plasmid without insert were used as negative control.

### Statistical analysis

The erythrocyte-binding assay results of the Fy^a+/b+^ and Fy^a+/b-^ erythrocytes (*n* = 4 for each group) were analysed using SPSS v.20 statistical software (IBM, Chicago, Illinois, USA). A Shapiro-Wilk test was used to determine normality. An independent t-test was used to compare the mean difference of Fy^a+/b+^ and Fy^a+/b-^ erythrocytes. The difference was considered significant when *P* < 0.05. Cohen’s d-test was used to determine the effect size.

## Results

**Fig. 1:**
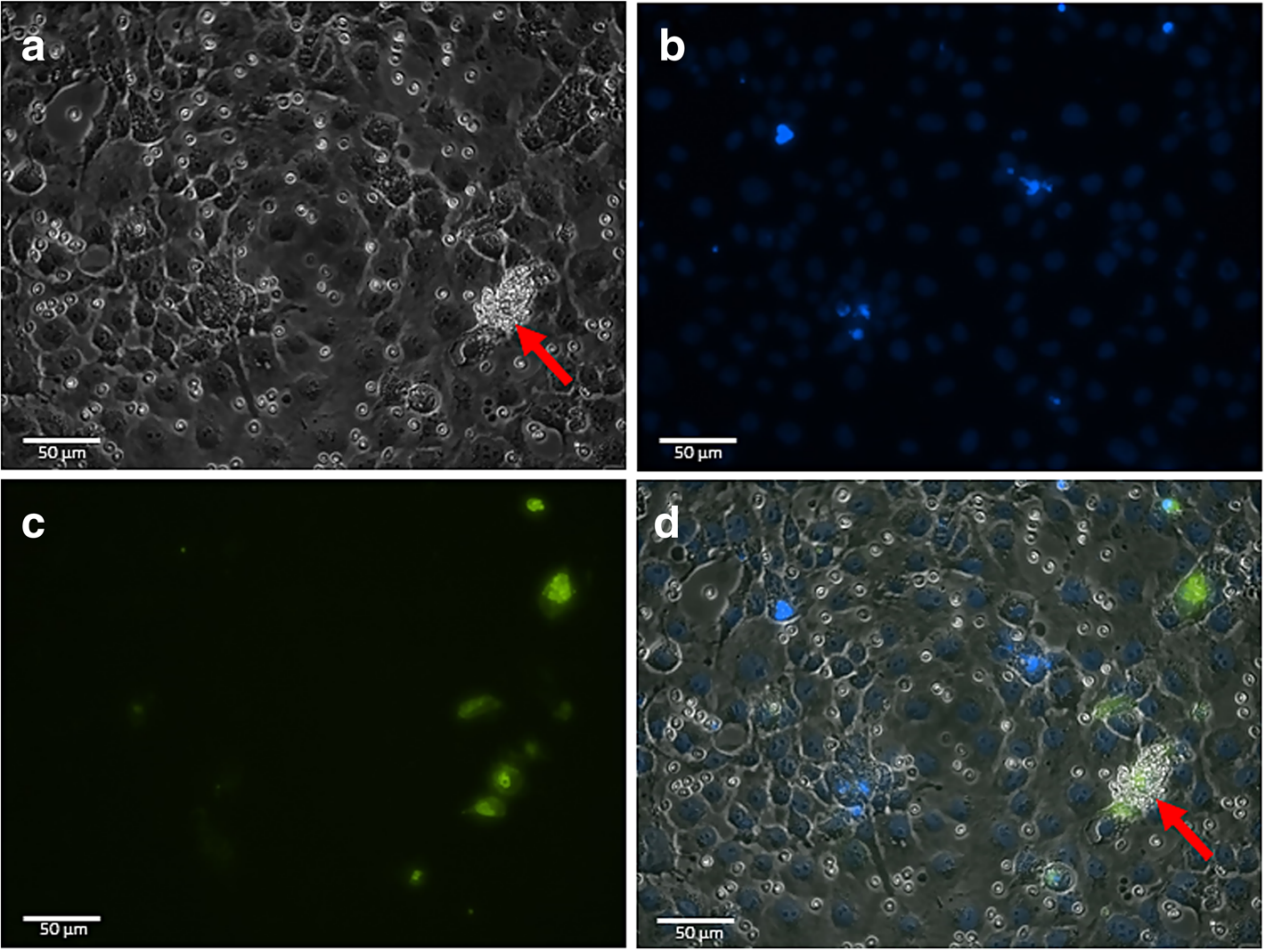
Erythrocyte-binding assay to determine binding activity of PkDBPαII to erythrocytes. **a** Rosette formation (red arrow) on PkDBPαII-pDisplay™ transfected COS-7 cell, with more than 50% of the cell surface covered by adherent erythrocytes. **b** Nuclei of COS-7 cells are stained blue with Hoechst dye. **c** Transfected COS-7 cells show green fluorescence indicating expression of GFP fluorescence tag and PkDBPαII. **d** Merged images of **a**, **b** and **c** showing the location of rosette, transfected cells and their nuclei

PkDBPαII was successfully amplified and cloned into eukaryotic expression vector pDisplay™. The recombinant plasmid was successfully transfected into COS-7 cells. Figure 1 shows PkDBPαII protein expression on COS-7 cells 48 h post-transfection (viewed with green fluorescence under a FITC-filter). In the negative control (COS-7 cells transfected with pDisplay™ plasmid without the *PkDBPαII* gene) no green fluorescence was observed. The Duffy grouping of blood donors’ erythrocytes was confirmed by ASP-PCR. The amplification product of 713 bp was yielded for both Fy^a^ and Fy^b^ alleles. Four samples each for phenotypes Fy^a+/b-^ and Fy^a+/b+^ were selected for the erythrocyte-binding assay.

The Shapiro-Wilk test showed that the rosette number scores for the four samples were normally distributed, hence an independent t-test was used to compare the mean difference between the binding level of Fy^a+/b-^ and Fy^a+/b+^ to PkDBPαII. The result revealed a significant difference in the binding level. The number of rosettes scored for Fy^a+/b+^ was 1.64-fold higher than that of Fy^a+/b-^ (155.50 ± 34.32 and 94.75 ± 23.16 rosettes, respectively; *t*_(6)_ = -2.935, *P* = 0.026) (Table 1). The Cohen’s d effect size value was 2.075, indicating the effect size was large and the means of the two groups differed more than 2 standard deviations. No rosette was observed in the negative control wells, indicating no binding of erythrocytes to the COS-7 cells transfected with empty plasmid.

**Table 1:** Erythrocyte-binding assays of PkDBPαII using Fy^a+/b-^ and Fy^a+/b+^ erythrocytes

Erythrocyte	Number of rosettes^a^	*t*-statistic	*P*-value	Cohen’s d^b^
Fy^a+/b-^ (*n* = 4) Fy^a+/b+^ (*n* = 4)	94.75 (± 23.16) 155.50 (± 34.32)	-2.935	0.026	2.075

^a^Number of rosettes [mean (± standard deviation)] seen in 30 fields at 200 × magnification

^b^Cohen’s d suggests that d ≤ 0.2 is considered a small effect size, 0.5 medium effect size and ≥ 0.8 large effect size

## Discussion

The Duffy antigen plays a key role in the invasion process of *P. knowlesi* and *P. vivax* into the human erythrocyte. The two major forms of the antigen, Fy^a^ and Fy^b^, have been shown to have different binding levels to *P. vivax* PvDBPII. Direct binding and antibody inhibitory binding experiments revealed decreased Fy^a^ binding efficiency to PvDBPII compared with Fy^b^. It has been implied that this decreased binding reduces the susceptibility of human individuals with an Fy^a^ blood group to *P. vivax* malaria. Furthermore, increased expression of Fy^b^ has been associated with an increase susceptibility to vivax malaria [8].

The only study that investigated PkDBP binding to Fy antigens was carried out almost 40 years ago [9]. The study used supernatant of *in vitro* cultured *P. knowlesi* as the source of PkDBP. The metabolically radiolabeled PkDBP from *P. knowlesi* culture supernatant that bound to erythrocytes was eluted and then analyzed by electrophoresis and autoradiography. The binding level was qualitatively determined by simply observing and comparing the intensity of PkDBP bands in the autoradiograph. In a related experiment in the study, the eluted radiolabeled PkDBP was used to probe membrane proteins from Fy^a^ and Fy^b^ erythrocytes that had been electrophoretically separated and transferred to nitrocellulose. Again, binding level was qualitatively determined by observing the intensity of bands in the autoradiograph.

In the present study, the binding level was quantitatively determined by counting the number of rosettes in the erythrocyte binding assay, and the difference in the mean number of rosettes obtained in the assays for Fy^a+/b+^ and Fy^a+/b-^ erythrocytes could be statistically tested. The results revealed that the mean number of rosettes obtained for Fy^a+/b+^ was significantly higher than that obtained for Fy^a+/b-^. Here, it can be surmised that the Fy^b^ antigen contributed to the higher binding of the Fy^a+/b+^ erythrocyte to PkDBPαII compared with the Fy^a+/b-^ erythrocyte, a feature which has also been observed in the binding of erythrocytes to PvDBPII [8]. This common feature can be explained by the fact that PkDBPαII and PvDBPII are orthologues which have similar critical domain and amino acid residues (Tyr94, Asn95, Lys96, Arg103, Leu168 and Ile175) for binding with Duffy antigen [14]. The difference in binding capacity of Fy^b^ and Fy^a^ to the parasites’ Duffy binding protein, on the other hand, is likely attributed to the substitution of Asp42 in Fy^b^ to Gly42 in Fy^a^ [7].

Cross-sectional studies on Duffy antigen groupings in Brazil [15], Iran [16] and India [17] revealed a higher prevalence of Fy^a+/b+^ in vivax malaria patients compared to healthy donors. It is believed that expression of the Fy^a^ and Fy^b^ genes in conditional heterozygote enhances infection by *P. vivax*, thus rendering Fy^a+/b+^ individuals more prone to infection. The question that now arises is: could the same could be true for knowlesi malaria? Hence, the next quest is to determine the prevalence of Duffy antigen phenotypes and knowlesi malaria in Malaysia and its surrounding countries.

## Conclusions

The erythrocyte-binding assay has provided a simple approach to quantitatively determine the binding level of PkDBPαII to the erythrocyte Duffy antigen. In this assay, PkDBPαII displayed higher binding to Fy^a+/b+^ erythrocytes than to Fy^a+/b-^ erythrocytes. Future studies need to be carried out to investigate whether the Fy^a+/b+^ phenotype is associated with increased risk of acquiring knowlesi malaria.

## Abbreviations

ASP-PCR: allelic specific PCR
CO_2_: Carbon dioxide
DARC: Duffy antigen receptor for chemokines
DMEM: Dulbecco’s Modified Eagle’s medium
DNA: Deoxyribonucleic acid
FBS: Fetal bovine serum
FITC: Fluorescein isothiocyanate
GFP: Green fluorescent protein
PBS: Phosphate-buffered saline
PCR: Polymerase chain reaction
PkDBP: *Plasmodium knowlesi* Duffy binding protein
PvDBP: *Plasmodium vivax* Duffy binding protein

## References

[1] Singh B, Kim Sung L, Matusop A, Radhakrishnan A, Shamsul SS, Cox-Singh J (2004). A large focus of naturally acquired *Plasmodium knowlesi* infections in human beings. Lancet..

[2] Moyes CL, Henry AJ, Golding N, Huang Z, Singh B, Baird JK (2014). Defining the geographical range of the *Plasmodium knowlesi* reservoir. PLoS Negl Trop Dis..

[3] Iwagami M, Nakatsu M, Khattignavong P, Soundala P, Lorphachan L, Keomalaphet S (2018). First case of human infection with *Plasmodium knowlesi* in Laos. PLoS Negl Trop Dis..

[4] Millar SB, Cox-Singh J (2015). Human infections with *Plasmodium knowlesi* zoonotic malaria. Clin Microbiol Infect..

[5] Cowman AF, Tonkin CJ, Tham WH, Duraisingh MT (2017). The molecular basis of erythrocyte invasion by malaria parasites. Cell Host Microbe..

[6] Singh AP, Ozwara H, Kocken CH, Puri SK, Thomas AW, Chitnis CE (2005). Targeted deletion of *Plasmodium knowlesi* Duffy binding protein confirms its role in junction formation during invasion. Mol Microbiol..

[7] Tournamille C, Le Van Kim C, Gane P, Cartron JP, Colin Y (1995). Molecular basis and PCR-DNA typing of the Fya/Fyb blood group polymorphism. Hum Genet..

[8] King CL, Adams JH, Xianli J, Grimberg BT, McHenry AM, Greenberg LJ (2011). Fy(a)/Fy(b) antigen polymorphism in human erythrocyte Duffy antigen affects susceptibility to *Plasmodium vivax* malaria. Proc Natl Acad Sci USA..

[9] Haynes JD, Dalton JP, Klotz FW, McGinniss MH, Hadley TJ, Hudson DE (1988). Receptor-like specificity of a *Plasmodium knowlesi* malarial protein that binds to Duffy antigen ligands on erythrocytes. J Exp Med..

[10] Michon P, Fraser T, Adams JH (2000). Naturally acquired and vaccine-elicited antibodies block erythrocyte cytoadherence of the *Plasmodium vivax* Duffy binding protein. Infect Immun..

[11] Cheng Y, Wang Y, Ito D, Kong DH, Ha KS, Chen JH (2013). The *Plasmodium vivax* merozoite surface protein 1 paralog is a novel erythrocyte-binding ligand of *P. vivax*. Infect Immun..

[12] Lim KL, Amir A, Lau YL, Fong MY (2017). The Duffy binding protein (PkDBPαII) of *Plasmodium knowlesi* from Peninsular Malaysia and Malaysian Borneo show different binding activity level to human erythrocytes. Malar J..

[13] De Silva JR, Lau YL, Fong MY (2014). Genotyping of the Duffy blood group among *Plasmodium knowlesi*-infected patients in Malaysia. PLoS One..

[14] Singh SK, Hora R, Belrhali H, Chitnis CE, Sharma A (2006). Structural basis for Duffy recognition by the malaria parasite Duffy-binding-like domain. Nature..

[15] Cavasini CE, de Mattos LC, Couto AA, Couto VS, Gollino Y, Moretti LJ (2007). Duffy blood group gene polymorphisms among malaria vivax patients in four areas of the Brazilian Amazon region. Malar J..

[16] Miri-Moghaddam E, Bameri Z, Mohamadi M (2014). Duffy blood group genotypes among malaria *Plasmodium vivax* patients of Baoulch population in southeastern Iran. Asian Pac J Trop Med..

[17] Monhaty SS, Singh KV, Fotedar R, Lakshminarayana J, Parihar R (2011). Prevalance of Duffy blood groups among the population of the desert region of India. J Rural Trop Pub Health..

